# Assessment, Decision, Adaptation, Production, Topical Experts-Integration, Training, and Testing (ADAPT-ITT) Framework to Tailor Evidence-Based Posttraumatic Stress Disorder Treatment for People With HIV to Enhance Engagement and Adherence: Qualitative Results from a Feasibility Randomized Controlled Trial

**DOI:** 10.2196/64258

**Published:** 2025-01-16

**Authors:** Cristina M Lopez, Angela D Moreland, Stephanie Amaya, Erin Bisca, Christin Mujica, Tayler Wilson, Nathaniel Baker, Lauren Richey, Allison Ross Eckard, Patricia A Resick, Steven A Safren, Carla Kmett Danielson

**Affiliations:** 1 Department of Psychiatry & Behavioral Sciences Medical University of South Carolina Charleston, SC United States; 2 Department of Psychological Science University of Arkansas Fayetteville, AR United States; 3 Department of Public Health Sciences Medical University of South Carolina Charleston, SC United States; 4 Department of Medicine Infectious Disease Section Louisiana State University Health Sciences Center New Orleans, LA United States; 5 Department of Infectious Diseases Medical University of South Carolina Charleston, SC United States; 6 Department of Psychiatry and Behavioral Sciences Duke University Medical Center Durham, AL United States; 7 Department of Psychology University of Miami Coral Gables, FL United States

**Keywords:** PTSD, HIV, adherence, minoritized populations, adaptation, evidence-based, treatment, engagement, posttraumatic stress disorder, stress, trauma, antiretroviral therapy, therapy, symptoms, acceptability, self-efficacy

## Abstract

**Background:**

Individuals with co-occurring posttraumatic stress disorder (PTSD) and HIV are at high-risk for negative HIV-related outcomes, including low adherence to antiretroviral therapy, faster disease progression, more hospitalizations, and almost twice the rate of death. Despite high rates of PTSD in persons with HIV (PWH) and poor HIV-related health outcomes associated with PTSD, an effective evidence-based treatment for PTSD symptoms in PWH does not exist.

**Objective:**

This study aimed to describe the adaptation and theater testing of an evidence-based intervention designed for people with co-occurring PTSD and HIV.

**Methods:**

The Assessment, Decision, Adaptation, Production, Topical experts-integration, Training, and Testing (ADAPT-ITT) framework guided the formative process used to modify an evidence-based PTSD treatment (cognitive processing therapy; CPT) to meet the unique needs of PWH experiencing PTSD. With the integration of Life-Steps for Medication Adherence (Life-Steps), the adapted protocol (CPT-Life-Steps for integration of adherence; CPT-L) targeted HIV-related stigma and HIV medication adherence within a trauma-informed framework. Theater testing was completed with 7 participants to evaluate acceptability of CPT-L for PWH. The qualitative data (N=54 recordings) used to evaluate and adapt CPT-L emerged from individual interviews conducted with participants after each therapy session as well as exit interviews conducted at posttreatment data collection.

**Results:**

After challenging stigma-related appraisals, participants expressed feeling less constrained by maladaptive thoughts. These shifts translated to increased self-efficacy with both HIV-related care and mental health.

**Conclusions:**

These results indicate that trauma-informed work with PWH should consider the impact of HIV on trauma-related stuck points, intersecting identities (including living with HIV), and challenging internalized stigma. Findings provide evidence that CPT-L is acceptable and effective in addressing internalized HIV stigma that impacts PTSD symptom maintenance and HIV treatment engagement.

**Trial Registration:**

ClinicalTrials.gov; NCT05275842; https://clinicaltrials.gov/study/NCT05275842?id=NCT05275842&rank=1

**International Registered Report Identifier (IRRID):**

RR2-10.1016/j.conctc.2023.101150

## Introduction

### Background

By 2019, an estimated 1,189,700 people in the United States were living with HIV [1]. Although people with HIV (PWH) face many unique psychosocial risk factors that impact health outcomes, trauma exposure and posttraumatic stress disorder (PTSD) have been consistently identified among the most prominent stressors for this population. The prevalence of PTSD among PWH, 30%-74% [2,3] far exceeds that of the general population, 3.9% [4]. Trauma history endorsed for *DSM-5-TR* (*Diagnostic and Statistical Manual of Mental Disorders, Fifth Edition, Text Revision*) criterion A, diagnostic information across the lifespan can include a wide range of adverse childhood experiences, interpersonal violence, survivors of natural disasters, combat, immigration-related trauma, HIV-related trauma if identified as traumatic by patient, and other events endorsed as inducing fear of serious harm to self or a loved one. Data suggest that traumatic events most often reported by PWH include interpersonal violence (physical, sexual, or psychological in nature), witnessing domestic violence or physical abuse as children, and seeing someone be seriously injured or killed [5]. This high comorbidity has important treatment implications as patients with co-occurring PTSD and chronic medical conditions report forgetting (41%) or skipping (24%) medications at significantly higher rates than patients without PTSD (29% and 13%, respectively) [6]. In addition, cognitive impairment and increased avoidant behavior experienced as a function of PTSD may contribute to lower levels of antiretroviral therapy (ART) adherence for PWH with a comorbid PTSD diagnosis [7]. For instance, PWH reported avoiding their ART because it was a reminder of their status [7] and of a traumatic life event [8], and they reported avoidance of clinic care visits due to fear of prejudice and discrimination from health care providers [9].

Notably, advances in ART have led to significant improvements in disease-related morbidity, mortality, and health outcomes for PWH. With consistent use, ART markedly slows disease progression and improves quality of life by suppressing viral load, which can potentially be reduced to undetectable levels. However, failure to reach near perfect (≥95%) adherence rates may be insufficient and detrimental to achieving viral suppression. More specifically, suboptimal ART adherence is strongly linked to viral proliferation [10,11], medication resistance [10,12], disease progression [13], and even death [14]. Unfortunately, the necessary 95% to 100% ART adherence rates are not easily achieved considering the multifaceted barriers that PWH must overcome to reach and maintain the recommended benchmark.

The stigmas attached to HIV, mental health, and minoritized identities are one such barrier. Stigmas can impact PWH through three primary mechanisms: (1) enacted stigma experienced in the form of actual rejection or discrimination by people and society; (2) anticipated stigma, which is the expectancy of discrimination and marginalization; and (3) internalized stigma or negative self-perceptions related to having the disease [15]. In addition, intersectional stigma [16,17], has been shown to differentially impact PWH, such that individuals with additional minoritized identities (eg, women, individuals that identify as sexual and gender minorities, and minoritized racial groups) report higher rates of stigmatization [18], and medication nonadherence [19], compared with those with majority-based identities. Furthermore, stigmatization has also been associated with multiple social determinants of health, such as low education level, poverty, lack of access to health insurance, incarceration, and non-US country of origin [18]. In turn, stigmatization can manifest in significant health disparities including poorer health outcomes, elevated PTSD symptoms due to reinforcement of avoidant coping behaviors, and delayed enrollment in HIV care until significantly ill [18-22].

Theoretical models jointly attribute the observed health disparities to the presence of “minority stress” and the absence of “social safety” among PWH, especially those who experience intersectional stigma. Specifically, the minority stress model [23] proposes that heightened vulnerability to health problems results from the cumulative burden of psychological stress experienced in response to social stigmatization (ie, minority stress). In effect, chronic exposure to stigma may lead individuals to be more susceptible to the development of posttraumatic distortions about the world (eg, enacted stigma) and oneself (eg, internalized stigma) following a traumatic event [15,23]. In addition, stigma may reinforce avoidant coping behaviors that are commonly associated with PTSD (eg, lack of trust in new relationships like with a new provider; not taking medication because it reminds them of a negative thought or feeling, etc). In other words, minority stress can impact mental health by reinforcing maladaptive beliefs associated with PTSD and emotional disorders after traumatic or difficult experiences [24]. These findings emphasize the importance of addressing stigma within evidence-based psychosocial interventions as a means of reducing distress and paving the way for improvements in HIV medication adherence and health outcomes. Expanding on the minority stress model, social safety theory posits that, in the absence of social safety (ie, reliable social connection and protection), stigmatized PWH routinely navigate the world in a state of maintained vigilance that adversely impacts immune system response [25]. Threats in the social environment are thought to trigger physiological stress responses, including chronic inflammation, that can impair immunological function [25]. Thus, social safety theory suggests that targeting maladaptive beliefs (eg, internalized stigma) and interpersonal patterns (eg, avoidance) may amplify social safety, and improve health outcomes by extension, for trauma exposed PWH [25].

### Current Study

Due to the distinct connection between HIV and PTSD, it is important that evidence-based treatments be tailored to address both trauma symptoms and barriers to HIV care for PWH. Although effective interventions to address HIV medication adherence and PTSD are widely available, there are no established integrated approaches that address the dual issues of cooccurring HIV with PTSD and related health disparities. In addition, no research to date has examined the critical question of whether HIV outcomes can be improved among the large number of PWH with comorbid PTSD by treating PTSD symptoms to maximize benefits of health promotion interventions. To address this tremendous void in the field, we propose the integration and evaluation of 2 empirically supported interventions that have been successfully used to improve PTSD- and HIV-related outcomes in this population, namely cognitive processing therapy (CPT) [26], and Life-Steps for Medication Adherence (Life-Steps) [27].

Using the Assessment, Decision, Adaptation, Production, Topical experts-integration, Training, and Testing (ADAPT-ITT) model, a framework for adapting HIV-related evidence-based interventions [28], CPT and Life-Steps were integrated to encompass HIV-related stigma and ART adherence within the scope of PTSD treatment. Upon adapting the intervention for PWH and PTSD, the resulting model (CPT-L) was theater-tested with treatment-seeking members of the intended population. More specifically, maladaptive beliefs influenced by exposure to trauma can interfere with important correlates of health, including a participant’s motivation to remain ART adherent [29], access social support [30], and establish trusting relationships with medical care providers [31]. To tackle these psychosocial factors, CPT-L includes cognitive restructuring components introduced weekly through modified worksheets and handouts reflecting topics that are more relevant to the realities of living with co-occurring HIV and PTSD. Acknowledging that these health conditions are experienced within a broader personal and cultural context, CPT-L also placed emphasis on engaging in discussion and exploration of the participants’ intersecting identities (eg, HIV status, racial, ethnic, sexual, and gender identity discrimination) [32]. This study presents qualitative findings of feedback solicited to assess reactions to the new intervention and elicit critiques that will guide future efforts to refine and tailor CPT-L.

## Methods

### Treatment Adaptation

Aligned with the ADAPT-ITT model, the CPT-L adaptation process will comprise 8 phases: assessment, decision, adaptation, production, topical expert, integration, training, and testing; and it has primarily been used in the context of HIV [33]. Findings in the current manuscript reflect the first 4 completed phases of the ADAPT-ITT protocol. During the initial step (assessment) of the ADAPT-ITT framework, the team consulted with the Community Advisory Board (CAB), comprising of constituents with lived experience (with PTSD or HIV), Ryan White clinic personnel (eg, providers, case managers, and staff), peer advocates, and community-based mental health providers. CAB members and topical experts were provided with a review of the extant literature to assess the scope of the problem and whether it merited development of new evidence-based treatments to address comorbid PTSD and HIV. Consideration was given to studies on mental health disparities in PWH as well as conflicting evidence about the efficacy of existing treatment modalities for HIV and co-occurring mood disorders. Evidence suggests that although existing psychosocial interventions can be effective in treating depression among PWH, symptom improvement does not translate consistently to improvements in HIV-specific markers (eg, ART adherence and viral load) [34]. Therefore, the team heeded CAB feedback and determined that adaptation was necessary to attend to the unique and unmet needs of this population.

In the second step (decision), the CAB and team’s selection of CPT among the available evidence-based PTSD interventions was driven by its flexibility to integrate HIV-specific content and CPT’s established training model for successful dissemination [35,36]. CPT, is an evidence-based treatment shown to be effective for reducing posttraumatic distress, including PTSD and depressive symptoms, among civilian and military adult populations with complex histories of exposure to traumatic events [37,38]. The protocol is designed for 12 sessions, but the length of treatment can be adapted based upon the patient’s needs [39]. In terms of structure, after centering the first half of treatment on dysfunctional cognitions about the worst traumatic event, the focus shifts to maladaptive beliefs that highlight the following 5 areas commonly impacted by trauma: safety, trust, power and control, esteem, and intimacy. CPT has been shown to reduce trauma-related self-blame and guilt [40,41] and may be particularly beneficial for PWH because of the focus on “stuck points.” Stuck points are negative, inaccurate, and unhelpful beliefs about yourself, others, or the world that occur as a result of trauma. Maintaining these stuck points prevents the individual from recovering from PTSD. The goal of CPT is to assist the patient in identifying stuck points related to their traumatic event, and then challenge these stuck points or negative thinking patterns through cognitive restructuring [42]. The process of cognitive restructuring is used to identify alternative, more accurate, and more helpful thoughts through Socratic questioning (ie, open-ended questions from the therapist that stimulates the patient to question current beliefs as flawed). In addition, the broad scope covered under the 5 themes allows for the flexibility to address internalized stigma related to HIV and marginalized identities. Indeed, although it was not originally developed as an HIV intervention, CPT has shown potential in promoting HIV risk-reduction [43].

Adaptation focused on modifying CPT to bolster its effectiveness with HIV medication adherence and related health outcomes. The CAB’s decision to incorporate Life-Steps for medication adherence [27], into CPT was made based on studies showing its efficacy and successful integration with various mental health treatment modalities [27,41]. Life-Steps is a one-session intervention using cognitive behavioral strategies, problem-solving, and motivational interviewing to promote adherence. The modified intervention (CPT-L) used the single-session adjunct to establish baseline HIV and ART psychoeducation and problem-solving skills. CPT-L later leveraged this foundational knowledge by incorporating continuous reminders of the benefits of remaining ART adherent and restructuring maladaptive thoughts that posed barriers to HIV care. The integration of the Life-Steps component also addressed health literacy and concerns about ART through tailored explanations about the need for consistent adherence, psychoeducation on the co-occurrence of PTSD and HIV, and rehearsal of adaptive health behaviors (eg, recognizing barriers to adherence and using problem-solving strategies to confront them). In the context of the larger study aims, CAB members chose Life-Steps as the control condition to ensure that participants assigned to the study arm would receive benefit as well.

The third step (adaptation) was completed through a larger pilot trial. The protocol was theater-tested with members of the target population, namely PWH and co-occurring PTSD. Aligned with the ADAPT-ITT model, reactions and feedback about the intervention were elicited from participants and stakeholders and include the qualitative findings presented here. Currently, CPT-L has a drafted manual (stage 4 of the ADAPT-ITT model) and plans to continue the adaptation process with topical expert feedback are underway. Refer to Table 1 for CPT-L protocol modifications based on ADAPT-ITT steps 1-4 that have been completed. Next stages of the project include an iterative process of refining the manual by eliciting comments and suggestions from our CAB, clinical team, peer advocates and other key constituents (step 5–Topical expert feedback), integration of that feedback in the CPT-L manual (step 6–Integration), and training of personnel to deliver the adapted intervention (step 7).

**Table 1:** Components and modifications for a tailored, evidence-based PTSD treatment for people with HIV (cognitive processing therapy- Lifesteps): adaptations informed by a qualitative study using the empirically supported ADAPT-ITT framework.

Sessions		Intervention component	CPT-L^a^ refinements
1		Life-Steps; begin overview of PTSD^b^ and CPT^c^	Psychoeducation about connection between HIV status, stigma, and trauma; how held identities impact trauma and HIV experiences; assign additional impact statement assigned if trauma and receiving news about HIV status are separate events
2-3		Finding maladaptive HIV beliefs	Identify beliefs related to internalized stigma through HIV-related impact statement; use HIV-related examples in context of ABC worksheets
4-5		Processing the index event	Distinguish trauma event processing from receiving news about HIV status; processing stigma from learning of HIV status; use HIV-related examples in context of challenging questions worksheets
6-7		Learning to self-challenge	Use HIV-related examples in context of challenging beliefs worksheets
8-10		Trauma themes (safety, trust, and power-control)	Regain “control” of health by challenging stigma-related stuck points; adaptation of Trust Star Worksheet to include status disclosure
11-12, 12+		Esteem, intimacy, and facing the future	Challenging stigma-related non-adaptive cognitions, duty to warn review for clinician
**Delivery adaptations**			
	Format change	Variable length	Could use additional “stressor sessions” to discuss stigma-related stuck points^39^ in CPT^c^ variable length format
	All	Adherence self-monitoring	In addition to risk assessment in CPT^c^, CPT-L^a^ includes ART^d^ adherence monitoring
	All	Trauma, stigma, and HIV connection	Continually developing the connection between HIV status and trauma; discuss impacts of intersectionality on HIV and trauma experience, as appropriate
	All	Practice assignments	Therapist to encourage challenging both trauma-related and HIV-related stuck points between sessions (if separate events)

^a^CPT-L: CPT-Life-Steps for integration of adherence.

^b^PTSD: post-traumatic stress disorder.

^c^CPT: cognitive processing therapy.

^d^ART: antiretroviral therapy.

Often, when adaptations of evidence-based treatments are attempted, the process generally ends there. However, a critical element of the ADAPT-ITT model includes conducting a test of the full intervention (step 8). While the involvement of stakeholders across multiple phases of the adaptation processes helps ensure that the adaptation resonates with the community and reflects their needs, the last and final phase of the ADAPT-ITT model determines that the adapted intervention has met the goal of creating positive and incremental impact for a specific population. Thus, subsequent steps of the adaptation process for CPT-L include a larger trial to evaluate the final product in a rigorous and systematic way. The submitted project proposal for the large-scale testing of CPT-L also includes an aim focused on implementation science and CPT-L intervention scale up, an important aspect to the team to remain aligned with the feedback from the CAB members.

### Participants and Recruitment

Participants were recruited from the Infectious Disease Department’s Ryan White Clinic at the academic medical center. A full description of the study procedures is reported elsewhere [44]. All protocols were approved by the institutional review board at the affiliated university. The study’s program coordinator recruited participants during their regularly scheduled HIV care clinic appointments and used a “cold call” list to contact individuals with HIV outside of these appointments. The study’s purpose, methods, and compensation were discussed with participants, and if they were interested in participating, the initial contact assessment was completed. This initial assessment included completing a shortened version of the PTSD checklist for *DSM-V* (*Diagnostic and Statistical Manual of Mental Disorders* [Fifth Edition]; PCL-5), as well as the Montreal Cognitive Assessment (MoCA). If participants scored at least 3 out of 5 on the shortened PCL-5, indicating possible PTSD, and scored within the mild or moderate range on the MoCA assessment (ie, no indication of severe cognitive impairments), then the consent process was completed. Consented participants then completed the clinician administered PTSD scale for DSM-5 to determine if they met criteria for PTSD. Participants who met criteria for PTSD were able to continue through the study. Additional eligibility criteria included (1) age 18 years or older enrolled in a Ryan White clinic; (2) able to speak, read, and write English; (3) meet at least one of the following HIV care criteria: (a) diagnosed with HIV in last 3 months; (b) detectable viral load in last 12 months; (c) failed to show up for or missed 1 or more HIV care appointments in the past 12 months; (d) last HIV care visit was more than 6 months ago; and (e) self-reporting less than 90% ART adherence in the past 4 weeks.

CPT-L was completed individually with a licensed professional counselor and was offered virtually and in person, depending on the participant’s location and preference. All participants of the theater testing phase were provided with the standard CPT workbook, as well as modified worksheets specific to CPT-L (eg, Stuck Point log with examples of HIV related stuck points, modified ABC, Challenging Question, Challenging Beliefs worksheets with examples using HIV related stuck points, and a Trust Star geared toward aspects of HIV status disclosure).

As part of a larger pilot trial using theater testing for the first stage, 7 patients completed end of session interviews at the end of each CPT therapy session and exit interviews at the end of protocol (54 total session recordings; number of sessions per patient ranged from 1 to 12; M=7). Race and ethnicity included African American (n=5) and non-Hispanic White (n=2). Participants’ ages ranged from 23-64 years. There were 4 males and 3 females, and participants identified as gay (n=2), bisexual (n=2), or heterosexual (n=3). Index traumas included life threatening illness or injury (n=1), physical assault (n=1), transportation accident (n=1), sexual assault or rape (n=2), and other unwanted sexual experiences (n=2).

### Procedures

Patients participated in end of the session adaptation questions following each session and intervention exit interviews that explored strengths and weaknesses of CPT-L, including feedback on adaptations of CPT to people with HIV and suggestions for improvement in the adaptation before the large scale randomized controlled trial. The end of session adaptation form consisted of 2 questions that asked for participants to provide a rating from 1 to 10 (1=not at all and 10=very much): “How much did the materials covered in today’s session address aspects of people living with HIV?” and “How much did the handouts reflect your experiences living with HIV and PTSD?” Participants were also asked to provide open-ended feedback on these questions and additional information regarding how useful the materials were and how they will use the skills taught in therapy in their everyday lives. In addition, exit interviews were conducted at the end of treatment. Exit interviews consisted of 6 primary questions and 13 follow-on questions (Table 2) that took place in 2022, in English, over videoconference and lasted approximately 5-15 minutes. Participants were compensated US $25 for completion of each exit interview.

**Table 2:** Exit interview questions completed by patients with HIV and comorbid PTSD at the end of a qualitative study exploring acceptability and relevance of a tailored, evidence-based PTSD treatment for people with HIV (cognitive processing therapy-Lifesteps).

Primary question	Follow-up question
Tell me about the mental health treatment or counseling you have received before	What did you like? What did you dislike? What was missing? What could have been more helpful? How did you feel about the overall experience?
Tell me your thoughts about the 12-session trauma and adherence-based intervention that you just completed over the past 6-8 weeks	Was the length of treatment problematic? Was it beneficial to discuss the topic of your trauma or stressful life experience? Was the intervention beneficial to discuss and process thoughts about your HIV status? What reservations do you have about recommending this treatment to be available clinically? What changes, if any, do you think would need to be made to trauma treatment to make it most helpful, relevant and appropriate for PWHa? Do you think that the same trauma treatment that is used with other groups would be equally helpful for PWH^a^? Why or Why not?
Did you feel that the CPT-L^b^ intervention specifically addressed HIV-related topics?	How do you feel that your experience of the trauma is different or unique than a survivor who is not living with HIV?
What coping behaviors for reducing your traumatic stress symptoms did you learn from participating in CPT-L^b^?	Are these coping strategies different than the ones you used before CPT-Lb? How so?
What additional insights did you gain about the identities you hold?	Did the examples and discussion included in the intervention resonate with the different identities you hold?
What concerns do you have if a friend were asked to participate in this study?	—^c^

^a^PWH: persons with HIV.

^b^CPT-L: CPT-Life-Steps for integration of adherence.

^c^Not applicable.

### Data Analysis

All qualitative recordings (end of session adaptation questions and exit interviews) were transcribed verbatim. Following transcription, content from the qualitative recordings were analyzed using a systematic thematic analysis within a grounded theory approach [45,46]. A “ground-up” approach involves an iterative process of establishing codes, grouping the codes into key concepts, and organizing the concepts into broader themes. NVivo (version 12) software was used to organize data and conduct the qualitative analyses. This software package facilitates coding, storage, retrieval, and analysis of large amounts of transcribed data. The codebook was created in a collaborative, inductive, and iterative process. All qualitative recordings were coded by a primary coder (second author) and 25% of recordings were double coded for interrater reliability. Both coders have expertise in qualitative coding of mental health research; neither coder had any interaction nor relationships with participants. Meetings between coders were held to reach a consensus on inconsistent codes for each transcript.

The qualitative researchers were careful to ensure data saturation in the current study given that only 7 participants were included. In qualitative research, there are 5 components that influence data saturation which include predetermined codes and themes, sample size, relevancy of research subjects, number of research tools, and length of data collection sessions [47]. When analyzing the responses from the 7 participants, the qualitative experts determined that data saturation was reached based upon the themes directly mapping onto the questions asked in the interview, previous research showing that saturation can be reached in approximately 10 interviews, that all participants in this study were patients receiving CPT-L and thus, were the most relevant to answer the questions about treatment, and the length of data collection certainly increased the chance of early saturation in this study given that 54 interviews were collected over the data collection period.

### Ethical Considerations

The study was approved by the Medical University of South Carolina Institutional Review Board for Human Research (Pro00106801). Before engaging in the study and collecting data, each participant was required to give written informed consent, either in person or virtually using the Medical University of South Carolina’s institutional review board approved REDCap (Research Electronic Data Capture) e-Consent. Within the consent process, research staff discussed with potential participants that the results of the research study, including interviews, may be published and information would not be identifiable. The procedures also clarified that all information obtained from participants would be confidentially maintained. Research staff received training from the principal investigator on the importance of confidentiality and had weekly supervision meetings with the principal investigator ensuring strict compliance with the Human Subjects Protection plan. Additional measures for protecting privacy and confidentiality also included assigning each participant a unique project identification number. The codes that linked the name of the participant and the study ID number were kept confidential by the principal investigator in a secured, locked cabinet. The secure servers that housed the data files collected from the web applications (REDCap) during were located at MUSC in Secure Sockets Layer 128-bit encrypted servers behind firewalls. Participant confidentiality was masked in all data files by the use of project identification numbers rather than personal information.

After all the relevant information was reviewed verbally with trained research staff, participants signed the document (either virtually or in person) and the signed consent document was stored electronically (from e-consent or scanned in) in the REDCap database, which provides password protected, secure, web-based collection and management of research. Informed consent was conducted by members of the study team that received training through the University of Miami CITI training in both the Conduct of Human Subject Research and Good Clinical Practice, and were institutional review board–approved study personnel. Study data collected, including exit interviews, was conducted either in-person in the privacy of a clinic room, or remotely through telephone. Participants were informed when the interview recording began and ended and were instructed not to use the names of individuals or reveal any personally identifiable information. These audio files were deidentified within 24 hours and uploaded into the REDCap electronic study database to later be transcribed. Any files on a portable recording device were deleted following this upload, and only the project therapist, fidelity raters, and qualitative coders heard the content of the audio recordings. Participants were not compensated for interviews conducted at the end of each CPT-L session, but were compensated US $25 for end of study exit interviews.

## Results

### Descriptive Characteristics

Regarding the materials addressing aspects of people living with HIV, participants rated the materials very highly (mean 9.86, SD 0.93) and indicated that the handouts greatly reflected the experiences of living with HIV and PTSD (mean 9.71, SD 0.61).

### Qualitative Results

Four overarching themes, each with their own subthemes, emerged from the semistructured interviews: (1) overall strengths of CPT-L, (2) overall suggested improvements of CPT-L, (3) helpful handouts/activities/materials, and (4) ways that patient would like to incorporate skills learned from CPT-L.

#### Theme 1: Overall Strengths of CPT-L

Most patients discussed overall strengths of CPT-L, with the main subthemes emerged including that patients generally felt materials were relatable and relevant to HIV, information was relevant to HIV, learned tools unique and specific to trauma related to HIV, discussed topics never addressed before, reported general improvements in well-being and hope, and learned a different (less negative) view of HIV status following CPT-L engagement. In discussing that they generally felt materials were relatable and relevant to HIV, one patient reported, “It’s making perfect sense and I think it’s really helpful,” and another patient noted, “[CPT-L] is very sensible. It’s pragmatic, it’s logical. I wish I had this a long time ago.” Several patients discussed that the model was very relevant to HIV, making statements such as, “everything was covered. HIV, on what living with HIV is,” and “Oh yes, those would be concerns that people living with HIV would have.”

All patients discussed at some point in the exit interviews that they learned tools to address relevant issues. For example, 1 patient stated,

I felt increasingly as I participated that it was giving me some tools to live more – well, to be more whole and not feel as constrained by like stuck points in a way.

Another patient discussed,

I wish I’d had these tools a very long time ago ‘cause I could have probably worked through some things more successfully.

Relatedly, several patients reported that they discussed topics in CPT-L that they have never discussed before, including

It’s honestly the first time since that sexual abuse that I even tried to tackle this,” and “PTSD as far as a self-recognized condition is kind of new to me. So there’s still kind of a process of exploration about that.

In describing that they now have a different view of their HIV status following engagement in CPT-L, 1 patient stated,

I don’t see HIV as a threat to me as much as I once did.

Another patient described,

Knowing that it’s not my fault. That you know, I’m HIV and still can be successful.

Finally, most of the patients discussed that they felt general improvements in well-being and hope following CPT-L. One patient described,

I honestly feel a little more hopeful. I feel better about my decision about how to deal with this feeling of hopelessness. And I think that I’m finding that I put more reasonable weight on events than I used to and some of it is coming from this where I’m just not looking. I feel like I’ve got a prism if you will look at things and not necessarily have them so heavily weighted one way or the other.

Other patients discussed improvements in well-being and hope by stating,

I feel a little stronger about being able to take care of myself. Not just as far was physiologically with procedures but with my state of mind

and

I think [CPT-L] is increasing, you know, kind of my recognition of my place in life. And that I can improve things. You know? And you know, not feel so crestfallen about my place in the world.

#### Theme 2: Suggested Improvements in CPT-L

About half of the patients discussed suggested improvements in CPT-L, with the main subthemes including adding more HIV-relevant examples, to individualize the activities, and to clarify some of the handouts. Specifically, some patients who suggested adding more HIV relevant examples stated, “Is there a way to make the trust worksheets more reflective of HIV,” “There were like only 3 choices and they weren’t really talking about HIV,” and “The handouts might be better with advocate stories from people with HIV.” One patient who thought the activities could be more individualized mentioned, “I think that for me though because of my generation so to speak and some of the things I’ve seen while I’ve been HIV positive as well as had my own sexual orientation that those things might be, – they don’t touch on me so much.” Another patient stated, “I just think that some people will respond well to this method and other’s less so.” Finally, some patients discussed ways to clarify some of the handouts with statements such as, “Some of them were not self-explanatory altogether,” and “As far as like the terminology that maybe there’s something to you know actually define it.”

#### Theme 3: Helpful Handouts, Activities, and Materials

All 7 of the patients gave feedback on their perceived helpful handouts, activities, and materials during completion of CPT-L, with the main subthemes including handouts, challenging stuck points, and exposure activities. All patients made positive comments about the handouts, including general comments about the handouts such as

The handouts made me reflect on the things that I asked me to do and things that I need to let go of,

The handouts reflected someone living with both HIV and PTSD. They reflected a lot,

and

I think that they could equally be applied to a lot of other difficult circumstances people have. And I think with certain individuals that it might be more helpful for them to have something that’s tangible.

Several patients also made positive comments about specific handouts, including

I actually like this trust star so to speak. I think that it could actually be pretty useful just as an exercise about trust generally rather than specifically.

Almost all of the patients discussed that the challenging stuck points activities were helpful. Specifically, 1 patient noted,

To reflect on, you know, an issue and look at it from a lot of different facets in a lot of different ways,

and another mentioned,

It works. I mean it’s asking me directly to address the [stuck points] and to put things down and look at them and go through a systematic process. You don’t like activating them but it’s working.

In addition, most patients reported that the exposure techniques were very helpful in decreasing symptoms. One patient described,

Just talking about it. I mean, you let me trigger some things that I didn’t even think I was gonna think about, yeah?

#### Theme 4: Incorporating Skills Learned in CPT-L

Many of the patients described ways that they plan to incorporate skills learned in CPT-L moving forward, including setting different goals, engaging in different activities, practicing challenging thoughts and stuck points, being more aware of feelings, and improving self-acceptance and self-esteem. Regarding goals, many patients described that they plan to set different goals for themselves. One patient reported,

I wanna set different goals for myself and maybe think about how I’m doing things a little bit differently and as far as you know, making a more dive into this state.

Another patient stated some personal goals,

Not being in my PJs and taking a shower before session. And combing my hair.

In addition, a patient discussed a goal of consistently taking medication by stating,

I need to make sure I’m on my meds for the next three months to get shots and I hate shots, so this has helped me.

Relatedly, several patients described that they plan to engage in different activities following completion of CPT-L, including

I can start planning something. I will start with people from church” and “If I have to do at least one thing just for myself, not because I did anything to deserve it, or whatever right? I can do that.

Most patients described that they plan to practice changing thoughts and challenging stuck points following completion of CPT-L. Specifically, 1 patient reported,

I think that there’s a great likelihood that I’m probably going to filter what I think and how I react,

while another patient stated,

Reconstruct my perception or think about alternatives.

A few patients reported that they plan to be more aware of their feelings, as 1 patient stated,

Assessing feelings, and probably being more aware of them. And their impact on people around me.

Finally, several of the patients described that they would like to improve their self-acceptance and self-esteem following the completion of CPT-L. Specifically, 1 patient made a comment about self-acceptance and self-esteem including,

The way I think about what happened with my mother and trying to use it as a lens to help me have a better sense of self instead of looking for some reason why I’m the way I am. Just more acceptance of the way I am. Because I mean, I don’t feel like I’m altogether horrible.

Other patients described,

Try to think more positive about myself,

and

I can already see this has given me a construct with some new things that float into my mind. That are kind of self-judgments that I can, you know, have a better way of dealing with.

## Discussion

Recent years have seen a growing awareness of the need for trauma-informed, HIV treatment approaches as converging evidence suggests a reciprocal association between HIV and exposure to trauma. Having an HIV diagnosis is a significant predictor of physical and sexual victimization [48-49], which in turn has been associated with more engagement in HIV-risk behaviors, poorer physical functioning, increased symptomology, and higher rates of mortality among PWH [12,50-54]. Unfortunately, PWH with co-occurring PTSD must overcome several structural, psychosocial, and behavioral challenges with potentially devastating health consequences. These mutually reinforcing risk factors call for an integrated treatment approach [55]. Indeed, integrated interventions for cooccurring disorders have been found to increase client engagement with treatment and improve patient outcomes [56]. To address this unmet need, the current study introduced a modified treatment protocol incorporating gold standard, evidence-based practices used with trauma exposed populations CPT and PWH (Life-Steps) [27,57]. CPT was selected from similarly effective treatments for PTSD for its mechanism of change (ie, cognitive restructuring), which allows for the flexibility to simultaneously target maladaptive appraisals related to trauma, intersectional stigma, and HIV using a skills-based, modular format.

The resulting framework (CPT-L) leverages treatment components from each protocol to target factors known to contribute to PTSD and ART nonadherence. First, structural barriers, such as low access to (and understanding of) health education, can considerably impede adherence given the complexity of HIV medication regimens [58]; components of CPT-L addressing these documented barriers were well-received by participants in the current study. The integration of Life-steps with CPT provided explanations about consistent adherence and psychoeducation about the co-occurrence of PTSD and HIV. Generally, participants found that the information adapted from both protocols was well integrated and accurately reflected their lived experience. Findings suggest that demystifying co-occurring HIV and PTSD allowed participants to gain a sense of mastery over their own medical care, which then translated to actionable goals to increase and maintain higher levels of ART adherence. The observed patterns were consistent with studies supporting the effectiveness of education-based interventions on treatment adherence for PWH [59,60] but extended existing interventions by incorporating information on the impacts of PTSD. Although some participants lamented not having access to the information sooner, they also noted that it has contributed to a newfound self-awareness and sense of hope. Within the context of the roles of stigma and attitudinal barriers in delayed mental health services, the high patient acceptability of CPT-L are promising and warrant further testing to help improve access to trauma-focused care, both in the United States and abroad [61].

Another known factor contributing to PTSD and ART nonadherence that is addressed by CPT-L are maladaptive beliefs in a multicultural context. In addition to “traditional” (ie, more trauma-focused) maladaptive beliefs that are identified for cognitive restructuring in CPT, increased acceptability and relevance of CPT-L by PWH may be due to CPT-L’s emphasis on participants’ intersecting identities (eg, HIV status, racial, ethnic, sexual, and gender identity discrimination) to acknowledge and validate broader personal and cultural contexts that can affect health behaviors and management. This multicultural orientation opened a conversation about experiences of stigma (enacted and internalized) they may have experienced. Participants were then guided in processing intersectional stigma through a trauma-informed lens (ie, themes of safety, trust, power and control, esteem, and intimacy). For example, aligned with evidence of the association between disclosure of HIV status and increased social support [43], the “Trust Star Worksheet” was adapted to reflect disclosure as an important type of trust. The Trust Star is an activity completed within CPT to assist the patient in identifying aspects and levels of trust, and how this impacts the way the individual may trust themselves and others. While many individuals with PTSD view trust as an all or none concept, this activity assists the patient in seeing trust as multi-faceted. This in turn assists the patient in challenging extreme or exaggerated stuck points related to trust. Based on results from feedback on the qualitative study, the new conceptualization of the Trust Star in CPT-L included the participant’s trust in their own ability to navigate the process of disclosure of their status using their newly acquired tools (captured in the theme “different (less negative) view of HIV status”. Upon completing the protocol, participants recognized that much of the shame they experienced about their HIV status, victimization experiences, and intersecting identities was founded on flawed beliefs about the nature of the disease, the world, themselves, and others (captured in themes “practicing challenging thoughts and stuck points” and “improving self-acceptance and self-esteem”). Using thought challenging skills, participants developed more adaptive worldviews that fostered greater self-acceptance (especially regarding sexual orientation and gender identity), trust in others, and trust in themselves (captured in themes “practicing challenging thoughts and stuck points”). Consequently, they were better able to engage in self-care (eg, hygiene and medication adherence) and were more motivated to connect with their social support networks. From the perspective of a social safety theoretical framework, in removing inappropriate shame and internalized stigma that hinders social bonds, feedback from participants highlights CPT-L’s potential as an effective tool to decrease some of the deleterious health consequences that traumatized PWH experience as a function of social threat and disconnection [62].

Finally, PTSD-associated avoidance may underlie ART nonadherence such that the medications themselves may cue trauma memories and HIV-related stigma that deter consistent use. In other words, regardless of the reason (eg, forgetting vs choosing), it is possible that medication noncompliance is functionally an avoidant behavior used to escape distressing internal experiences for people struggling with co-occurring HIV and PTSD. In response, CPT-L features exposure as a core intervention component. Specifically, rather than the standard trauma narrative, participants are assigned 2 reflection statements recounting their index trauma and their experience with the news of their HIV status (unless HIV status is the index trauma). For many participants, the therapy setting was their first experience talking, or even deliberately thinking, about their index trauma and HIV status. They generally observed that actively approaching these topics allowed them to reorganize their thoughts and reframe their experiences. To their surprise, participants discovered that their broadened perspectives cultivated esteem and intimacy as they reflected on their relationships to themselves and others. These findings were consistent with evidence suggesting that disclosing trauma histories within the context of research or health care settings poses minimal risk of worsening symptoms, and in some cases can have secondary therapeutic benefit [63]. This data may help address misinformation and provider fears about “retraumatizing” PWH when screening for PTSD. In addition, participants were able to reconceptualize the cause and meaning of their index trauma and HIV status. Coupled with their fostered sense of trust in their own abilities, the shift away from self-blame and internalized stigma helped them regain a sense of control of their health and greater confidence in their ability to manage their own care.

While the intervention was largely well-received, several themes reflecting the need for modifications also emerged within the data. Participants primarily focused on improving handouts and worksheets stating that although the material was helpful, it was not intuitive, and the terminology was sometimes unclear. Several participants specified that they would benefit from additional examples pointedly touching on issues at the intersection of HIV and PTSD. Participants maintained that they would like to see self-acceptance and self-esteem for trauma-exposed PWH modeled through treatment materials (eg, including participant “success stories” and examples of adaptive alternatives to common stuck points).

Notwithstanding these limitations, CPT-L appears to be acceptable and relevant to PWH experiencing traumatic stress and merits further investigation as an integrated intervention for co-occurring PTSD and HIV. After challenging stigma-related stuck points, participants expressed feeling less constrained by maladaptive thoughts. These shifts translated to increased self-efficacy with both HIV-related care and mental health. Although limited in size and scope, the current study yielded preliminary support for the tolerability and effectiveness of the intervention. Findings highlight the importance of tailoring mental health treatment approaches to address trauma-related stuck points, intersecting identities (including living with HIV), and challenging internalized stigma. Such adaptations support goals for health outcomes (eg, ART adherence) by reducing the intensity of trauma-related distress and patterns of avoidance that interfere with HIV-treatment engagement. Future studies can expand on these efforts to complete the ADAPT-ITT process, including refining and testing CPT-L on a wider scale. Based on these qualitative results, integration and evaluation of strategies that foster social safety in mental health interventions for PWH warrants further attention.

## Abbreviations

ADAPT-ITT: Assessment, Decision, Adaptation, Production, Topical experts-integration, Training, and Testing
ART: antiretroviral therapy
CAB: Community Advisory Board
CPT: cognitive processing therapy
CPT-L: CPT-Life-Steps for integration of adherence
DSM-V: Diagnostic and Statistical Manual of Mental Disorders (Fifth Edition)
DSM-5-TR: Diagnostic and Statistical Manual of Mental Disorders, Fifth Edition, Text Revision
Life-Steps: Life-Steps for Medication Adherence
MoCA: Montreal Cognitive Assessment
PTSD: posttraumatic stress disorder
PWH: persons with HIV
REDCap: Research Electronic Data Capture

## Data Availability

The data generated for this study are not publicly available due to the sensitive nature of qualitative data and collection procedures. Recordings and transcripts of interviews are not publicly available given the confidentiality agreements with participants that were reviewed upon study enrollment.
